# Functional Status in Elderly Kidney Transplant Recipients: A Systematic Review Evaluating Physical Function, Frailty, and Cognitive Impairment as Predictors of Post-Transplant Outcomes

**DOI:** 10.3390/diseases13070229

**Published:** 2025-07-21

**Authors:** Hachem Araji, Yazan A. Al-Ajlouni, Jana Nusier, Walid Sange, Elie El-Charabaty, Suzanne El-Sayegh

**Affiliations:** 1 Department of Internal Medicine, Northwell, New Hyde Park, New York, NY 11040, USA; alajlouni.yazan@gmail.com (Y.A.A.-A.); wsange@northwell.edu (W.S.); 2 Faculty of Medicine, Jordan University of Science and Technology, Irbid 22110, Jordan; jnusier@gmail.com; 3 Department of Internal Medicine, Division of Nephrology, Northwell, New Hyde Park, New York, NY 11040, USA; eelcharabaty@northwell.edu (E.E.-C.); selsayegh@northwell.edu (S.E.-S.)

**Keywords:** elderly kidney transplantation, frailty, physical function, cognitive impairment, post-transplant outcomes

## Abstract

Background: The management of end-stage renal disease (ESRD) is undergoing a paradigm shift, with increasing emphasis on kidney transplantation as a preferred treatment modality for elderly patients (≥65 years), who constitute a substantial portion of new ESRD cases. Transplantation offers markedly superior survival and quality of life (QoL) advantages compared to dialysis for this demographic. Nevertheless, key determinants such as frailty, physical functionality, and cognitive function have emerged as critical predictors of post-transplant success. Despite their relevance, standardized methodologies for evaluating these parameters in transplantation candidacy remain absent. This systematic review examines the influence of frailty, physical functionality, and cognitive function on outcomes in elderly kidney transplant recipients. Methods: Adhering to PRISMA guidelines, a rigorous literature search was conducted across PubMed, CINAHL, Embase, PsycINFO, and the Web of Science for studies published up to October 31, 2024. Relevant studies focused on elderly transplant candidates and examined correlations between frailty, physical functionality, or cognitive function and post-transplant outcomes. The Newcastle–Ottawa Scale was employed to evaluate studies quality. Results: Seven studies met the inclusion criteria. Five explored physical functionality, demonstrating that better pre-transplant physical performance predicts enhanced survival. Two studies addressed frailty, utilizing the Fried frailty phenotype, and linked frailty to elevated mortality and diminished QoL recovery. Notably, no studies explored cognitive function in elderly kidney transplant candidates or recipients and its association with post-transplant outcomes, exposing a salient gap in the literature. The included studies’ varied methodologies, reliance on single time-point assessments, and exclusive focus on kidney transplant recipients restrict both comparability among studies and the generalizability of findings to the broader end-stage renal disease (ESRD) population. Conclusions: These findings underscore the profound impact of physical functionality and frailty on transplant outcomes in the growing elderly kidney transplant population, illuminating the necessity for standardized assessment protocols and targeted pre-transplant interventions. The critical gap in cognitive function research underscores a vital direction for future investigation. This research received no external funding. This review is registered with PROSPERO under registration ID CRD42025645838.

## 1. Introduction

The landscape of end-stage renal disease (ESRD) treatment is evolving, with kidney transplantation emerging as a critical option for elderly patients. In the United States, individuals over 65 account for more than three-quarters of incident ESRD cases; however, only about 26% of kidney transplant recipients in 2024 fall within this age group [1,2]. Recent policy changes, such as the ESRD Treatment Choices Model [3], have introduced financial incentives for dialysis providers to increase transplant referrals, potentially expanding the number of elderly patients being evaluated for transplantation [2].

Around the world, there is a similar increasing burden of CKD and ESRD among the elder population, with similar trends in European countries, including Germany [4,5]. Around 80,000 people in Germany alone undergo renal replacement therapy, with the highest proportion being older adults over the age of 65 [6,7]. These trends highlight the need for effective transplant evaluation protocols for elderly patients to improve outcomes in this population.

Kidney transplantation offers significant advantages over dialysis, including improved survival, enhanced quality of life (QoL), and lower long-term costs [1]. However, elderly transplant recipients face unique challenges. While transplantation often provides greater benefits than dialysis, the typically limited life expectancy in this population raises concerns regarding its impact on longevity, complications, and QoL improvements [8]. Unlike other solid organ transplants, kidney transplant programs do not impose a universal age cutoff; instead, candidates undergo individualized evaluations [9]. While transplant centers routinely assess cardiovascular, pulmonary, metabolic, and malignancy risks, there is increasing recognition that additional factors should be considered, given the significant phenotypic variation among elderly patients [8].

Current CKD management involves not only pharmacological intervention and dialysis but also technological innovations such as telemedicine, wearable dialysis monitors, and Al-based risk stratification models [10,11]. This helps provide personalized care and improve pre- and post-transplant outcomes. Moreover, the importance of nursing care in nephrology and dialysis has also significantly expanded. In addition to technical advances, relational and communication skills are essential in building patients’ trust to improve compliance and eliminating psychosocial barriers among elderly patients undergoing renal care [12].

Three key factors influencing post-transplant outcomes in older adults—frailty, cognitive impairment, and physical function—remain underexplored in pre-transplant evaluations. Frailty, a syndrome marked by reduced strength, endurance, and physiological reserve, heightens vulnerability to adverse outcomes and is highly prevalent in elderly patients with chronic kidney disease (CKD) [8]. Despite its clinical relevance, there are no standardized guidelines for integrating frailty into transplant eligibility decisions [13]. Similarly, cognitive impairment, a transitional state between normal cognition and dementia, is an emerging risk factor for poor outcomes in ESRD patients [14]. While dementia is considered a relative contraindication for transplantation, no established guidelines exist for assessing cognitive impairment in transplant candidates [14]. Physical function, defined as an individual’s ability to perform daily physical tasks, also plays a crucial role in transplant outcomes. Studies suggest that physical deconditioning is common among elderly renal patients and is associated with poor post-transplant recovery [15]. Despite growing interest in functional assessment tools, no standardized protocols exist for incorporating these measures into the transplant selection process [15].

A comprehensive pre-transplant evaluation should ideally predict post-transplant outcomes, particularly in terms of survival and QoL. However, no widely used predictive models currently exist to assess transplant candidacy in elderly patients [9]. This systematic review aims to address this gap by examining the role of frailty, cognitive function, and physical function in pre-transplant assessments. We will explore the current methods used to evaluate these factors in elderly kidney transplant candidates, assess their impacts on QoL and long-term survival, and review any reported post-transplant changes. Additionally, we aim to identify key considerations for integrating these factors into the transplant selection process. By investigating these elements, this review seeks to improve our understanding of their influence on transplant outcomes and inform future refinements in candidate selection for elderly patients being evaluated for kidney transplantation.

## 2. Methods

This systematic review was conducted in accordance with the Preferred Reporting Items for Systematic Reviews and Meta-Analyses (PRISMA) guidelines [16]. A comprehensive literature search was performed on 31 October 2024 across five major databases: PubMed, CINAHL, Embase, PsycINFO, and the Web of Science. Predefined keywords were used to identify relevant studies, as outlined in Table S1 of the Supplementary Materials. This review is registered with PROSPERO under registration ID CRD42025645838.

### 2.1. Search Strategy and Selection Criteria

A structured search strategy was developed using Boolean operators to refine and enhance the identification of pertinent studies across the selected databases. The specific search terms employed are detailed in Table S1 of the Supplementary Materials. No publication year restrictions were applied in the search strategy. Following the database search, all retrieved articles were imported into EndNote 20.0 reference management software, where duplicate entries were removed.

The eligibility of studies was assessed through a two-stage screening process. First, two independent reviewers screened the titles and abstracts to exclude irrelevant articles. Next, the full texts of the remaining studies were reviewed for inclusion based on predefined eligibility criteria. Disagreements between reviewers were resolved through discussion until a consensus was reached.

Studies were included if they met the following criteria: (1) focused on elderly patients (≥65 years) undergoing or being evaluated for kidney transplantation, (2) assessed frailty, physical function, or cognitive function in the context of kidney transplantation, (3) reported outcomes related to QoL, survival, or changes in frailty, physical function, and cognitive function post-transplant, (4) were observational studies, randomized controlled trials, or systematic reviews, and (5) were published in English. Studies were excluded if they (1) did not focus on elderly kidney transplant patients, (2) did not assess frailty, physical function, or cognitive function in relation to kidney transplantation, (3) failed to report relevant outcomes, (4) were case reports, commentaries, editorials, or non-peer-reviewed literature, (5) focused on other organ transplants or non-transplant interventions (e.g., dialysis-only studies), or (6) were duplicate publications without new analyses. The full inclusion and exclusion criteria are detailed in Table S2 of the Supplementary Materials. Table S4 presents the PRISMA 2020 checklist as it applies to our systematic review.

### 2.2. Data Collection and Presentation

A standardized data extraction form was developed to ensure consistency in collecting relevant information. The extracted data included bibliographic details, study objectives, participant characteristics, transplant evaluation methods, and reported outcomes related to frailty, physical function, and cognitive function. Specific attention was given to studies that examined changes in these factors post-transplant.

Data extraction was independently performed by one reviewer and reviewed by a second to ensure accuracy. The extracted information was synthesized and organized into tables to facilitate comparative analysis. One table was dedicated to studies focusing on pre-transplant assessments of frailty, physical function, and cognitive function, while another summarized the findings on post-transplant changes and their impacts on patient outcomes.

### 2.3. Quality Appraisal

The quality of the included studies was assessed using the Newcastle–Ottawa Scale (NOS), a validated tool for evaluating the risk of bias in observational studies [17]. The NOS evaluates studies across three domains—the selection of study groups, comparability of cohorts, and assessment of outcomes or exposures—assigning a maximum score of nine stars. Studies with higher scores were considered to have a lower risk of bias, whereas those with fewer stars indicated potential methodological limitations. Two independent reviewers conducted the appraisal, resolving any discrepancies through discussion or consultation with a third reviewer when necessary.

### 2.4. Ethics Approval and Role of Funders

As this study is based on previously published literature, ethical approval was not required. All included studies had undergone prior ethical review by their respective research teams. Additionally, this systematic review did not receive any external funding.

## 3. Results

### 3.1. Literature Search

**Figure 1:**
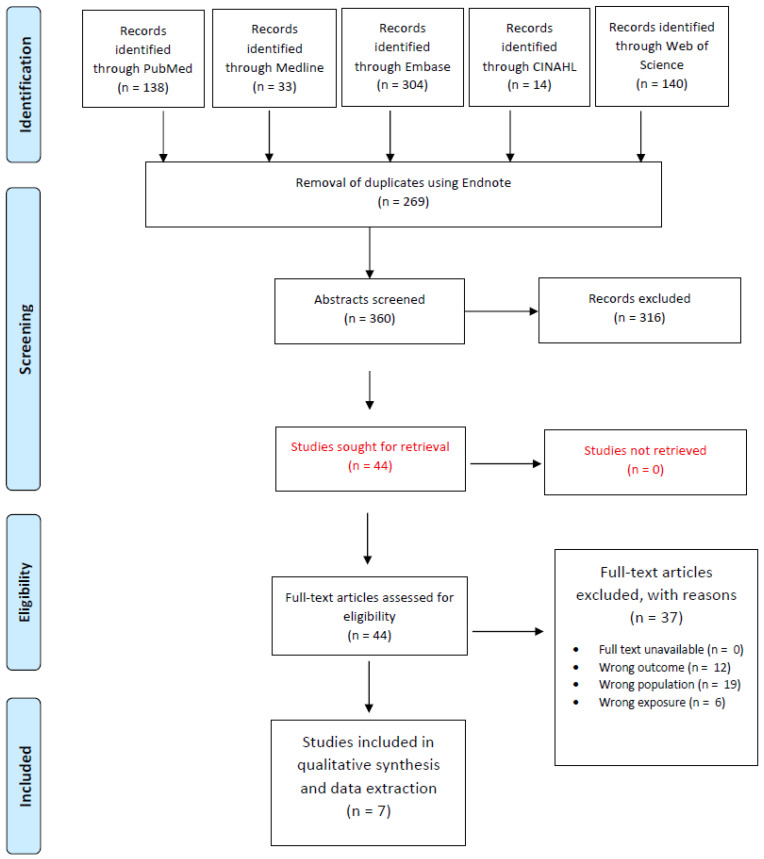
Preferred Reporting Items for Systematic Reviews and Meta-Analyses (PRISMA) study selection flow diagram outlining the literature review process when searching for articles on various databases.

Figure 1 outlines the study selection process for our systematic review. A comprehensive literature search across five databases—PubMed (*n* = 138), Embase (*n* = 304), Medline (*n* = 33), CINAHL (*n* = 14), and the Web of Science (*n* = 140)—yielded a total of 629 records. After removing 269 duplicates via EndNote, 360 unique records were screened based on titles and abstracts. This initial screening led to 44 full-text articles being assessed for eligibility. Following full-text evaluation, 37 articles were excluded due to reasons such as wrong outcome (*n* = 12), wrong population (*n* = 19), and wrong exposure (*n* = 6), resulting in 7 studies that were included in the final qualitative synthesis.

### 3.2. Quality Assessment

The quality assessment of the included studies, evaluated using the NOS [17], demonstrated an overall low risk of bias across most domains (Table S3, Figures S1 and S2). The highest-quality studies consistently met the criteria for selection, comparability, and outcome assessment, particularly in areas such as representativeness of the exposed cohort, selection of the non-exposed cohort, and ascertainment of exposure. However, one study had an unclear risk of bias in the design and analysis domain, indicating potential methodological concerns regarding confounding adjustment or study design robustness. Additionally, all studies adequately addressed follow-up duration and outcome assessment, ensuring reliable long-term evaluations of post-transplant outcomes.

### 3.3. Overview of Findings

#### 3.3.1. Physical Function and Post-Transplant Outcomes

Five studies [18,19,20,21,22] investigated the relationship between physical function and post-transplant survival in elderly kidney transplant recipients (≥65 years). These studies employed both retrospective [18,20] and prospective [19,21,22] cohort designs, with sample sizes ranging from 181 [21] to 26,721 [18] participants (Table 1).

Various methods were used to assess physical function (Table 2), including self-reported measures such as the Physical Function (PF) Subscale of the SF-36 questionnaire [20] and the Kidney Disease Quality of Life Short Form, version 1.3 (KDQOL-SF v1.3) [19,21]. Objective performance-based assessments included the Short Physical Performance Battery (SPPB) [22] and a classification of assistance levels [18]. Despite methodological differences, all five studies consistently demonstrated that better pre-transplant physical function was associated with lower post-transplant mortality. Studies utilizing objective assessments, such as the SPPB, provided stronger predictive value for survival compared to self-reported measures. Prospective studies further indicated that post-transplant changes in physical function correlated with long-term survival outcomes, emphasizing the need for continuous physical function assessment in elderly kidney transplant recipients.

The timing of assessments varied. In two studies by Tsarpali et al. [19,21], physical function was assessed pre-transplant and followed over time, with trajectories of functional decline post-transplant predicting mortality risk [21]. One study [21] reported that recipients with a poor functional trajectory had a 2.4-fold increased mortality risk, emphasizing the importance of tracking changes rather than relying on a single pre-transplant assessment. Nastasi et al. [22] found that recipients ≥65 years with SPPB impairment had a 27.1% five-year mortality rate, compared to 8.5% in unimpaired recipients. This suggests that interventions targeting lower extremity function before transplantation could improve survival.

Retrospective analyses using UNOS/OPTN data also reinforced the importance of physical function status. Reese et al. [20] demonstrated that the absolute difference in three-year mortality between the highest and lowest physical function quartiles was 14% in recipients ≥65 years, the largest discrepancy among all age groups. Brar et al. [18] similarly found that age itself was not predictive of survival, emphasizing that physical function status should be evaluated alongside age in transplant decision making.

Despite methodological differences, all studies consistently link higher pre-transplant physical function with improved outcomes (Table 3).

#### 3.3.2. Frailty as a Predictor of Transplant Outcomes

Two studies [23,24] examined frailty as a determinant of post-transplant outcomes in elderly recipients, employing the Fried frailty phenotype as the standardized assessment tool. Both studies followed a prospective cohort design, with sample sizes ranging from 443 [24] to 537 [23] participants. It is important to note that these studies [23,24] employed a modified scoring system for the Fried frailty criteria, differing from the original in keyways and potentially impeding direct comparisons of results with studies using the original scoring system. McAdams-DeMarco et al. [23] found that frailty was significantly associated with increased post-transplant mortality; frail recipients had significantly lower one-year survival rates (85.8%) compared to non-frail (97.5%) and intermediately frail (97.4%) recipients. This association was independent of age. The other study [24] evaluated the impact of frailty on health-related quality of life (HRQOL) post-transplantation, showing that younger frail recipients experienced a 5.33-point increase in physical HRQOL, whereas older frail recipients showed a 0.50-point decline (*p* = 0.02). Similarly, kidney disease-specific HRQOL improved by 12.55 points in younger frail recipients but only 5.17 points in older frail recipients (*p* = 0.04). In both studies, frailty assessments were performed only at the time of transplantation; no longitudinal assessment was conducted (Table 4).

#### 3.3.3. Cognitive Function and Transplant Outcomes

No studies in our review specifically examined cognitive function in elderly kidney transplant candidates or recipients and its association with post-transplant outcomes.

## 4. Discussion

This systematic review sought to evaluate the impact of physical function, frailty, and cognitive function on post-transplant outcomes in elderly kidney transplant recipients, highlighting assessment practices, their influence on quality of life and survival, and any changes observed after transplantation. To the best of our knowledge, this systematic review is the first to comprehensively assess the impact of physical function, frailty, and cognitive function on post-transplant outcomes in elderly kidney transplant recipients. The inclusion of only seven studies indeed reflects a genuine paucity of available research specifically addressing functional status and its association with post-transplant outcomes in the elderly kidney transplant population. This scarcity is not primarily attributable to overly stringent inclusion criteria but rather to the fact that most published studies did not conduct age-specific analyses or report outcomes separately for elderly recipients. As a result, the evidence base for this important subgroup remains limited. 

Our findings highlight that better pre-transplant physical function is associated with improved survival, whereas frailty increases mortality risk and affects post-transplant HRQOL. Despite these associations, assessments of physical function and frailty remain inconsistent across studies, and no research to date has examined the role of cognitive function in this population. Additionally, most studies relied on single time-point evaluations, limiting insights into longitudinal trends. These findings emphasize the need for standardized, evidence-based assessment protocols and targeted interventions to optimize transplant outcomes in older candidates. Although our review aimed to identify research focused on the optimal assessment of frailty, physical function, and cognitive function in elderly candidates prior to transplantation, we found that none of the included studies directly investigated this question.

### 4.1. Physical Function and Post-Transplant Outcomes

The results summarized in Table 2 and Table 3 highlight the prognostic significance of both self-reported and objective physical function assessments in predicting post-transplant survival for elderly kidney transplant recipients. Self-reported tools captured patient perspectives on daily activity limitations but were prone to subjective bias. The SPPB, an objective measure of lower extremity function, demonstrated stronger predictive value for post-transplant survival, although it does not account for upper body strength or endurance [22]. The consistent association between higher pre-transplant physical function and better post-transplant survival underscores the necessity of including such assessments in clinical evaluations of older transplant candidates.

Differences in the timing and frequency of assessments, as well as variability in tools used, present limitations for direct study comparisons. Most studies did not account for longitudinal changes in function beyond the first-year post-transplant, limiting insights into recovery patterns. Notably, none of the studies assessing physical function investigated its effect on quality of life after transplantation in the elderly population. Future studies should focus on standardized assessment time points and interventions that could enhance physical function before and after transplantation.

### 4.2. Frailty and Post-Transplant Outcomes

This review results reinforce frailty’s importance as a prognostic factor in post-transplant outcomes among elderly recipients. Not only was frailty linked to higher post-transplant mortality, but its impact was also found to be independent of chronological age [23], supporting the use of frailty as a distinct predictor during transplant evaluation. The findings on HRQOL suggest that while younger frail recipients may achieve improvements after transplant, older frail recipients are less likely to experience these benefits, indicating that physiological resilience may play a critical role in recovery. However, interpretation of these results is complicated by variations in frailty scoring methods, which may impede direct comparisons with studies using the original Fried criteria. An additional limitation is that frailty was assessed only at transplantation, thus the dynamic nature of frailty and its ongoing influence on outcomes remains unclear. These observations highlight the need for standardized frailty assessments and further longitudinal research to better inform risk stratification and optimize outcomes in elderly transplant candidates.

### 4.3. Cognitive Impairment and Post-Transplant Outcomes

Our review identified no studies that specifically investigated cognitive function in elderly kidney transplant candidates or recipients, and its association with post-transplant outcomes. A prospective study that did not exclusively focus on recipients aged >65 evaluated kidney transplant outcomes in recipients with cognitive impairment. In this study, cognitive impairment was assessed at the time of transplantation using the Modified Mini-Mental State (3MS) examination, and recipients with cognitive impairment were found to have a significantly increased risk of all-cause graft loss, defined as graft failure or mortality. Specifically, living donor kidney transplant recipients with any cognitive impairment had a 5.4-fold higher risk of all-cause graft loss compared to those without impairment, while deceased donor recipients with severe cognitive impairment had nearly a threefold increased risk. Notably, the prevalence of cognitive impairment was higher among older adults, with 22.9% of recipients aged 71–79 affected. These findings underscore that cognitive impairment—regardless of age—can substantially influence post-transplant outcomes, highlighting the importance of screening for and addressing cognitive deficits in kidney transplant candidates and recipients to improve survival and graft function [14].

In her comprehensive review [25], Golenia et al. highlighted that cognitive impairment is a prevalent but under-recognized issue in kidney transplant recipients; while validated tools like the Montreal Cognitive Assessment (MoCA) and Mini-Mental State Examination (MMSE) are widely used, the MoCA is preferred for its higher sensitivity in detecting mild deficits, yet challenges to routine cognitive evaluation include the lack of standardized, transplant-specific tools, confounding factors such as depression and immunosuppressive therapy, and resource constraints, highlighting the urgent need for future research to implement systematic screening, adjust for relevant comorbidities, and develop targeted interventions to better understand and address cognitive outcomes in this population [25].

### 4.4. Clinical and Public Health Implications

The findings of this review have direct implications for clinical practice, transplant policy, and public health initiatives, particularly in how frailty, physical function, and cognitive function are assessed and managed in elderly kidney transplant candidates.

The current pre-transplant evaluation process often prioritizes chronological age, yet our review highlights that physical function status and frailty are stronger predictors of long-term survival and quality of life. Integrating standardized, objective assessments of frailty (e.g., Fried frailty phenotype), physical function (e.g., SPPB), and, when possible, cognitive function could refine risk stratification and help transplant teams make individualized eligibility decisions.

Additionally, the evidence suggests that prehabilitation strategies targeting physical function and frailty may improve post-transplant outcomes [26]. Prehabilitation programs—including structured exercise regimens, strength training, and nutritional optimization—could enhance functional reserves before transplantation [27], potentially reducing postoperative complications and improving early HRQOL recovery. Given the association between frailty and increased post-transplant mortality, transplant centers may benefit from post-transplant rehabilitation programs aimed at maintaining or improving function after surgery.

Furthermore, longitudinal assessments of frailty and physical function should be incorporated into transplant follow-up care to identify patients at risk of functional decline. This would provide a more dynamic understanding of post-transplant recovery and facilitate timely interventions to mitigate poor outcomes.

From a public health standpoint, the lack of standardized frailty and physical function screening protocols in transplant evaluations represents a significant gap. Currently, there are no universal guidelines requiring transplant centers to assess frailty or physical function, leading to variability in candidate selection. Policymakers should consider incorporating these measures into national and international transplant allocation criteria, ensuring a more equitable and evidence-based approach to organ distribution.

Moreover, this review identifies a critical research gap: cognitive function remains unexamined in elderly kidney transplant candidates, despite its potential impact on medication adherence, rehabilitation participation, and long-term transplant success [25]. Cognitive impairment may influence the ability of patients to manage post-transplant care, making it essential for future research to evaluate cognitive screening tools alongside frailty and physical function assessments.

To improve patient outcomes and optimize resource allocation, prospective studies should explore how frailty, physical function, and cognitive function evolve post-transplant. These insights could guide the development of pre-transplant interventions, refine candidate selection criteria, and support post-transplant care models tailored to elderly recipients.

By integrating these assessments into clinical practice and transplant policies, healthcare systems can move toward a more comprehensive, patient-centered approach, ultimately improving transplant success rates, survival, and quality of life in elderly kidney transplant recipients.

### 4.5. Strengths and Limitations

This systematic review provides a comprehensive evaluation of frailty, physical function, and cognitive function in elderly kidney transplant recipients, synthesizing evidence on their assessment methods, prognostic significance, and impact on post-transplant outcomes. A key strength is the inclusion of both self-reported and objective physical function measures, allowing for a nuanced comparison of different assessment tools. Additionally, this review is the first to systematically highlight the absence of cognitive function studies in this population, emphasizing an important research gap. By adhering to PRISMA guidelines and using the Newcastle–Ottawa Scale for quality assessment, this review ensures methodological rigor, reducing bias in study selection and synthesis.

However, several limitations must be acknowledged. The included studies exhibited substantial heterogeneity in assessment methods and timing of evaluations, limiting direct comparisons and the ability to conduct a meta-analysis. Additionally, most studies relied on single time-point assessments, preventing insights into longitudinal changes in frailty, physical function, or post-transplant recovery trajectories. Furthermore, all included studies exclusively examined kidney transplant recipients, a cohort that has already undergone rigorous medical selection, resulting in a sample population with superior baseline health and functional capacity compared to the ESRD population. This selection bias may underestimate the true impact of functional status on post-transplant outcomes, potentially limiting the generalizability of findings to the broader elderly ESRD population considering transplantation.

The characteristics of the included studies’ population limit the generalizability of our findings. Since all included studies focused on an older population who had been selected for renal transplant, they represent a healthier and high-functioning subgroup of the larger ESRD population. This introduced selection bias, as patients with several comorbidities and a low functional status are unlikely to be added to the transplant list. Therefore, the association between frailty, physical function, and outcomes may not fully report the risk encountered by a more vulnerable population with ESRD. This should be kept in mind while applying the findings of this study to the general elder ESRD population on the renal transplant waiting list.

The exclusion of non-English studies may have introduced publication bias, and while we employed a broad search strategy, some relevant studies may not have been captured. Lastly, this review is limited by the absence of studies assessing cognitive function, a critical factor for post-transplant adherence, rehabilitation, and long-term outcomes, underscoring the need for further research in this area.

### 4.6. Future Research

Future research should focus on standardizing the assessment of frailty, physical function, and cognitive function in elderly kidney transplant candidates to improve risk stratification and patient selection. Prospective, multicenter studies with repeated assessments are needed to evaluate longitudinal changes in these factors and their impact on post-transplant outcomes. Additionally, interventional trials exploring the benefits of prehabilitation programs—including structured exercise, nutritional support, and cognitive training—could help optimize transplant success and long-term survival. Given the complete lack of studies assessing cognitive function, future research should investigate its role in post-transplant adherence, rehabilitation, and overall patient outcomes, integrating cognitive screening into transplant evaluations. Lastly, predictive models incorporating frailty, physical function status, and cognitive assessments should be developed to refine transplant decision making and guide personalized care strategies for elderly recipients.

From a clinical perspective, there is an urgent need for the integration of standardized protocols into routine pre- and post-transplant care. Simple tools such as the Fried frailty phenotype and the Short Physical Performance Battery (SPPB) [28] can be integrated and adopted in transplant facilities to support care planning and risk stratification. Moreover, around 30–60% ESRD patients are underassessed for cognitive impairment [29,30] despite its significant impact on treatment compliance and rehabilitation engagement. Implementing the use of cognitive screening tools such as the MoCA or Mini-Cog can be useful for early diagnosis and a personalized approach.

It is imperative to implement a multidisciplinary care model with nephrologists, geriatricians, physical therapists, dietitians, and neuropsychologists to address the complex needs of elderly patients undergoing renal transplant. Pre- and post-transplant rehabilitation can prove to be effective among these patients to improve functional capacity, enhance recovery, and improve patient-centered outcomes [31].

## 5. Conclusions

This systematic review highlights the critical role of frailty, physical function, and cognitive function in predicting post-transplant outcomes in elderly kidney transplant recipients. While better physical function is consistently associated with improved survival, frailty significantly increases mortality risk and affects post-transplant quality of life. Despite their clinical relevance, assessment methods remain inconsistent, and cognitive function has been entirely overlooked in this population. These gaps hinder the ability of current practices to completely address the comprehensive needs of elderly candidates for transplant. The findings highlight the need for standardized evaluation protocols, incorporating objective measures such as the SPPB and Fried frailty phenotype, to improve transplant decision making. Furthermore, prehabilitation strategies and post-transplant rehabilitation programs may offer opportunities to enhance functional reserves and long-term outcomes. The integration of cognitive screening and assessment is essential into clinical workflow to identify the patients at risk earlier and to guide a personalized timely intervention. Future research should prioritize longitudinal studies, cognitive assessments, and predictive modeling to refine patient selection and optimize transplant care for elderly recipients. By integrating these factors into clinical practice, transplant centers can move toward a more personalized, evidence-based approach that maximizes survival and quality of life for the growing elderly kidney transplant population.

## Figures and Tables

**Table 1:** Characteristics of studies evaluating physical function.

Author(s)	Year	Country	Study Design	Sample Size (Kidney Transplant Recipients)	Age Range	Mean Age	% of Recipients Were Aged ≥65 Years	% Gender Distribution (% Women)	Number of Participants on Dialysis	Time on Dialysis	Participants Not Yet on Dialysis at the Time of Evaluation	Median Follow-Up Time Post-Transplant
Reese et al. [20]	2014	U.S.	observational study (retrospective cohort study)	10,875		50	14	37	10,875	>1 years	0	1631 days
Brar et al. [18]	2021	U.S.	observational study (retrospective cohort study)	26,721	65 to 96		100	37	21,427		5226	
Nastasi et al. [22]	2018	U.S.	observational study (prospective, two-center, longitudinal study)	719	18.7 to 86.0 yrs	51.6 (SD = 14.2)	20	38	581		138	2 years
Tsarpali et al. [19]	2022	Norway	observational study (prospective, single-center, longitudinal study)	192	65 to 84 yrs	72.1 (SD = 4.1)	100	31	145	median = 27.5 months (17.6–43.2 months)	47	4.6 years
Tsarpali et al. * [21]	2022	Norway	observational study (prospective, single-center, longitudinal study)	181	65 to 84 yrs	72	100	31		median = 27.4 months (17.3–43.2 months)		4.9 years

US = United States; SD = standard deviation; yrs = years; * different study by the same first author published in the same year.

**Table 2:** Physical function assessment methods.

Author(s)	Year	Physical Function Assessment Method	Benefits	Limitations
Reese et al. [20]	2014	Physical Function (PF) Subscale of the Medical Outcomes Study Short Form-36 (SF-36) questionnaire	SF-36 has been validated for use in elderly patients and dialysis patients	PF scale is a subjective, self-reported measure
			Continuous measure that classifies patients over a spectrum of severity	
Brar et al. [18]	2021	Level of assistance (total assistance, moderate assistance, no assistance)	A straightforward categorization of physical status	A classification system is relatively crude
Nastasi et al. [22]	2018	Short Physical Performance Battery (SPPB)	Objective assessment of lower extremity function	Focuses on lower extremity function
			Well-validated physical assessment tool	May not capture the full spectrum of physical function
			Test takes approximately 5 to 10 min to complete	
Tsarpali et al. [19]	2022	Kidney Disease Quality of Life Short Form, version 1.3 (KDQOL-SF v1.3)	Relatively straightforward to administer and score	All HRQOL data are self-reported and may be subjected to bias
Tsarpali et al. * [21]	2022		Captures the patient’s perspective on their physical function and QoL	

QoL = quality of life; HRQOL = health-related quality of life; * different study by the same first author published in the same year.

**Table 3:** Main findings in studies evaluating physical function.

Author(s)	Year	Primary Outcome	Primary Exposure	Main Finding for Subgroup >65 Years of Age
Reese et al. [20]	2014	Patient survival after transplantation	Pre-transplant Physical Function (PF) score	Functional status predicted survival across all age groups, but recipients aged ≥65 years had the greatest risk difference between low and high PF quartiles
Brar et al. [18]	2021	Patient survival after transplantation	Assistance level, classified into 3 groups	Patient survival was significantly higher in recipients who needed no assistance and lowest in patients in need of total assistance
Nastasi et al. [22]	2018	Post-kidney transplant mortality	Short Physical Performance Battery (SPPB) score	Compared to younger unimpaired recipients, older impaired recipients (≥65 years) had a 2.60-fold higher risk of post-KT mortality
Tsarpali et al. [19]	2022	Patient survival after transplantation	Health-related quality of life, with a focus on the physical function component	A PF score ≤ 60 was associated with a 2-fold increase in mortality risk
Tsarpali et al. * [21]	2022	Patient survival after transplantation	Longitudinal HRQOL trajectories during the first post-KT year, particularly PF domain	Older recipients who experienced a poor PF trajectory during the first post-KT year had a 2.4 times higher mortality risk compared to those with a good PF trajectory

QoL = quality of life; HRQOL = health-related quality of life; KT = kidney transplant; * different study by the same first author published in the same year.

**Table 4:** Characteristics of studies evaluating frailty.

Author(s)	Year	Country	Study Design	Sample Size (Kidney Transplant Recipients)	Age Range	Mean Age	% of Recipients Were Aged ≥65 Years	% Gender Distribution (% women)	Number of Participants on Dialysis	Time on Dialysis	Participants Not Yet on Dialysis at the Time of Evaluation	Median Follow-Up Time Post-Transplant
McAdams-DeMarco et al. [23]	2015	U.S.	observational study (prospective, single-center, longitudinal study)	537		53 (SD = 14.0)		40				2.7 years
McAdams-DeMarco et al. [24]	2018	U.S.	observational study (prospective, two-center, longitudinal study)	443	19.9 to 86.0 yrs	52 (SD = 14.1)		37	379	Median = 3.26 years	64	7.7 months

US = United States; SD = standard deviation; yrs = years.

## Data Availability

The data analyzed in this systematic review were obtained from previously published studies, as cited within the manuscript. All relevant data are available in the referenced sources.

## Supplementary Materials

The following supporting information can be downloaded at: https://www.mdpi.com/article/10.3390/diseases13070229/s1, Table S1: Search Strategy and Keywords Used Across Databases (PubMed, Embase, CINAHL, PsycINFO, and MEDLINE); Table S2: Inclusion and Exclusion Criteria for Study Selection in the Systematic Review; Table S3: Results of study quality appraisal of the included studies using Newcastle–Ottawa quality assessment scale for cohort studies; Figure S1: Graphical Representation of Quality Assessment Scores for Included Studies Using the Newcastle–Ottawa Scale; Figure S2: Summary of Risk of Bias Across Included Studies Using the Newcastle–Ottawa Scale.
